# Unveiling cancer risk in ANCA-associated vasculitis: result from the Turkish Vasculitis Study Group (TRVaS)

**DOI:** 10.1007/s11739-024-03577-9

**Published:** 2024-03-29

**Authors:** Emre Bilgin, Tuba Demirci Yıldırım, Bahar Özdemir Ulusoy, Tahir Saygın Öğüt, Murat Karabacak, Öznur Sadioğlu Çağdaş, Reşit Yıldırım, Deniz Can Güven, Cansu Akleylek, Elif Ediboğlu, Muhammet Emin Kutu, Duygu Özgür, Rıza Can Kardaş, Ertuğrul Çağrı Bölek, Güllü Sandal Uzun, Zehra Özsoy, Emine Sarıyıldız, Gizem Ayan, Berkan Armağan, Abdulsamet Erden, Levent Kılıç, Funda Erbasan, Fatma Alibaz-Öner, Ebru Aşıcıoğlu, Ayten Yazıcı, Nazife Şule Bilge, Hamit Küçük, Selda Çelik, Cemal Bes, Servet Akar, Neslihan Yılmaz, Timucin Kaşifoglu, Ayse Cefle, Haner Direskeneli, Veli Yazısız, Ömer Dizdar, Ahmet Omma, Fatoş Önen, Ömer Karadağ

**Affiliations:** 1 Faculty of Medicine, Division of Rheumatology, Hacettepe University, Ankara, Turkey; 2 Hacettepe Vasculitis Research Center, Ankara, Turkey; 3 Faculty of Medicine, Division of Rheumatology, Dokuz Eylul University, İzmir, Turkey; 4 Division of Rheumatology, Ankara City Hospital, Ankara, Turkey; 5 Faculty of Medicine, Division of Rheumatology, Akdeniz University, Antalya, Turkey; 6 Faculty of Medicine, Division of Rheumatology, Marmara University, Istanbul, Turkey; 7 Faculty of Medicine, Division of Rheumatology, Kocaeli University, Kocaeli, Turkey; 8 Faculty of Medicine, Division of Rheumatology, Eskişehir Osmangazi University, Eskişehir, Turkey; 9 Division of Medical Oncology, Elazığ Fethi Sekin City Hospital, Elazığ, Turkey; 10 Faculty of Medicine, Division of Rheumatology, Demiroğlu Bilim University, Istanbul, Turkey; 11 Faculty of Medicine, Division of Rheumatology, Katip Çelebi University, İzmir, Turkey; 12 Division of Rheumatology, Bakırköy Sadi Konuk City Hospital, Istanbul, Turkey; 13 Division of Rheumatology, Başakşehir Çam Sakura City Hospital, Istanbul, Turkey; 14 Faculty of Medicine, Division of Rheumatology, Gazi University, Ankara, Turkey; 15 Faculty of Medicine, Division of Rheumatology, Marmara University, Istanbul, Turkey; 16 Faculty of Medicine, Division of Medical Oncology, Hacettepe University, Ankara, Turkey

**Keywords:** ANCA-associated vasculitis, Granulomatous polyangiitis, Cancer, Rituximab, Cyclophosphamide, Lung cancer, Head–neck cancers

## Abstract

To investigate cancer incidence in patients with ANCA-associated vasculitis (AAV), compare it with the age/sex-specific cancer risk of the Turkish population, and explore independent risk factors associated with cancer. This multicenter, incidence case–control study was conducted using the TRVaS registry. AAV patients without cancer history before AAV diagnosis were included. Demographic and AAV-related data of patients with and without an incident cancer were compared. Standardized cancer incidence rates were calculated using age-/sex-specific 2017 Turkish National Cancer Registry data for cancers (excluding non-melanoma skin cancers). Cox regression was performed to find factors related to incident cancers in AAV patients. Of 461 AAV patients (236 [51.2%] male), 19 had incident cancers after 2022.8 patient-years follow-up. Median (IQR) disease duration was 3.4 (5.5) years, and 58 (12.6%) patients died [7 with cancer and one without cancer (log-rank, *p* = 0.04)]. Cancer-diagnosed patients were older, mostly male, and more likely to have anti-PR3-ANCA positivity. The cumulative cyclophosphamide dose was similar in patients with and without cancer. Overall cancer risk in AAV was 2.1 (SIR) ((1.3–3.2), *p* = 0.004); lung and head-neck [primary target sites for AAV] cancers were the most common. In Cox regression, male sex and ≥ 60 years of age at AAV diagnosis were associated with increased cancer risk, while receiving rituximab was associated with decreased cancer risk. Cancer risk was 2.1 times higher in AAV patients than the age-/sex-specific cancer risk of the Turkish population population, despite a high rate of rituximab use and lower dose of cyclophosphamide doses. Vigilance in cancer screening for AAV patients covering lung, genitourinary, and head–neck regions, particularly in males and the elderly, is vital.

## Introduction

Anti-neutrophil cytoplasmic antibody (ANCA)-associated vasculitis (AAV) is an umbrella term including granulomatosis with polyangiitis (GPA), microscopic polyangiitis (MPA), and eosinophilic granulomatosis with polyangiitis (EGPA) [1]. The incidence and prevalence of these diseases exhibit geographical variations [2]. Depending on the subtype of AAV, there can be varying degrees of involvement in virtually any organ and tissue, predominantly the upper and lower respiratory tract, genitourinary system, and skin [1].

The relationship between cancer and AAV has been investigated in patients from various geographical regions with distinct genetic backgrounds. A meta-analysis published in 2015 combining six studies examining cancer incidence in AAV patients has determined the overall cancer incidence to be (pooled SIR) 1.74 (1.37–2.21) [3] and revealed an increase in non-melanoma skin, leukemia, and bladder cancers. The increased risk of cancer in AAV patients has often been attributed to the use of high-dose cyclophosphamide in the literature [3]. Considering the genetic complexity of cancer and AAV and their geographical differences, data from different ethnicities and geographies are necessary. On the other hand, following the recent demonstrations of its efficacy and safety, rituximab-based therapies have emerged as game changers in treatment practice.

In this study, we aim to investigate the incidence of cancer in AAV patients from Turkey with no previous cancer before the AAV diagnosis in the rituximab era, compare this incidence with the general Turkish population, and assess factors associated with cancer development in the Turkish AAV patients using the prospective Turkish Vasculitis Research Group (TrVAS) database.

## Patients and methods

### Study design

This incidence case–control study was conducted by the Turkish Vasculitis Study Group (TRVaS) registry [4].

### Data source and group of interest

TRVaS is a prospective, web-based registry of the Turkish Vasculitis Study Group established in 2020 [5]. In general, data regarding all incidents and prevalent cases with all types of primary vasculitis are recorded in TRVaS. Regarding AAV, a baseline assessment form including patient and disease characteristics was recorded for all patients. For this current study, all participating centers were asked to complete the prespecified data form related to ‘‘cancer’’ for patients diagnosed with any subtype of AAV by the end of July 2022.

Patients with any subtype of AAV (granulomatosis with polyangiitis [GPA], microscopic polyangiitis [MPA], eosinophilic granulomatosis with polyangiitis [EGPA] or unclassified AA-vasculitis) according to any of the following: the treating physician, 1990 ACR Wegener or Churg–Strauss classification criteria, or EMA algorithm and complete prespecified data form related to ‘‘cancer’’ were included in the current study. Among these patients, those with a history of malignancy before and at the time of AAV diagnosis and those with non-melanoma skin cancer or benign or non-invasive tumors (in the past or current) were excluded from the final analysis.

### Variables

i. Related to ANCA-associated vasculitis
  Date of birth, sex, date of AAV diagnosis, AAV subtype, ANCA status [enzyme-linked immunoassay (ELISA) and indirect immunofluorescence assay (IIFA)], sites of involvement, Birmingham Vasculitis Activity Score (v3) at baseline, and revised five-factor score were recorded. Immunosuppressive treatments were recorded as ‘ever or never used’ for the period between AAV diagnosis and cancer diagnosis in patients with cancer and between AAV diagnosis and the last visit in patients without cancer.
ii. Related to cancer
  Date of cancer diagnosis, type, and extensity (local, locally advanced, or metastatic) of cancer, smoking status (ever or never), and survival status (date of death was recorded for deceased patients) were recorded.

### Statistical analysis

Statistical analysis was performed using the SPSS software (v25.0; IBM Corporation, Armonk, NY, USA). The descriptive analysis was expressed as either median, interquartile range (IQR) for quantitative variables, or number (percentage) for categorical variables.

Distribution and univariable comparison of baseline demographics, disease characteristics, and treatment options used during the disease course of AAV patients with or without cancer were explored via the Kaplan–Meier survival estimates, and possible factors associated with cancer occurrence were investigated using the log-rank test. The Mann–Whitney *U* test was used to compare the quantitative variables between the two groups. The factors identified in univariable analysis (p < 0.20, taking into account the correction for multiplicity and clinical relevance) were further entered into the Cox regression analysis, with the backward selection, to determine independent predictors of cancer occurrence. The proportional hazards assumption and model fit were assessed using residual (Schonfeld and Martingale) analysis. To compare cancer incidences, age- and sex-specific standardized incidence rates (SIR) for the overall group, according to sex and individual cancer types, were calculated. For each participant, patient-year follow-up duration was calculated: starting from the date of AAV diagnosis to the date of cancer diagnosis, death, or last follow-up visit, whichever was the first. The observed number of cases is all individuals diagnosed with invasive cancer (except for non-melanoma skin cancer) on follow-up after the diagnosis of AAV. The expected number of cases is the total number of patients that would have been reported to the cancer registry within the same follow-up period and calculated from the 2017 Turkish National Cancer Registry (TNCR) data [6]. To compare the rates of cancers, incidence rates (IR) (per 1000 patient-years) and incidence rate ratios (IRR) for different subgroups (according to AAV subtype, ANCA status, renal and lung involvement, smoking status, age at diagnosis, and immunosuppressive drugs ever being used) were calculated. To calculate the SIR, IR, IRR, and the 95% confidence interval (CI), OpenEpi v3.01 software was used. Type-1 error lower than 0.05 was considered statistically significant.

Our study complied with the Helsinki Declaration and was approved by the Hacettepe University ethical committee (Approval number: GO 19/1088).

## Results

A total of 476 AAV patients were included in the study. Fifteen patients were excluded from the final analysis (8 had cancer before AAV diagnosis, 3 had colon cancer diagnosis during AAV workup, 3 had basal cell carcinoma, and 1 had non-invasive cancer). The final data set comprised 461 AAV patients (19 with cancer).

312 (67.7%) patients had GPA, 76 (16.5%) had EGPA, 52 (11.3%) had MPA, and 21 (4.6%) had unclassified AAV. The median (IQR) disease duration was 3.4 (5.5) years, 6.4 (7.7) years in AAV patients with cancer, and 3.4 (5.3) years in those without cancer (*p* = 0.013). The median (IQR) duration between AAV and cancer diagnosis was 46 (83) months. The total follow-up duration was 2022.8 patient years. AAV patients with cancer were older at AAV diagnosis compared to those without cancer (median, IQR) (61.3 [28.2] vs. 49.7 [22.3], *p* = 0.037). The male sex was predominant (94.7% vs. 49.3%, log-rank *p* < 0.001), and smoking (ever) was also more prevalent in this group (70.6% vs. 39.4%, log-rank *p* = 0.006). (Table 1).

**Table 1:** Distribution and time-dependent univariable comparison of demographic and disease characteristics of AAV patients with and without cancer

Variable	All patients (*n* = 461)	AAV patients with cancer (*n* = 19)	AAV patients without cancer (*n* = 442)	*P* value
Male, *n*(%)	236 (51.2)	18 (94.7)	218 (49.3)	< 0.001
Age at AAV diagnosis, years, median, (IQR)	49.8 (22.9)	61.3 (28.2)	49.7 (22.3)	0.037°
AAV disease duration*, months, median (IQR)	3.4 (5.5)	6.4 (7.7)	3.4 (5.3)	0.013°
Smoking (*n* = 403)				0.006
Never	239 (59.3)	5 (29.4)	234 (60.6)	
Ever	164 (40.7)	14 (70.6)	152 (39.4)	
AAV subtype				0.53
GPA	312 (67.7)	16 (84.2)	296 (67.0)	
EGPA	76 (16.5)	1 (5.3)	75 (17.0)	
MPA	52 (11.3)	1 (5.3)	51 (11.5)	
Unclassified	21 (4.6)	1 (5.3)	20 (4.5)	
ANCA IFA				0.77**
cANCA	227 (49.2)	12 (63.2)	215 (48.6)	
pANCA	123 (26.7)	3 (15.8)	120 (27.1)	
Negative	84 (18.2)	4 (21.2)	80 (18.1)	
Unavailable	27 (5.9)	0	27 (6.1)	
ANCA ELISA				0.03**
PR3	189 (41.0)	11 (63.6)	178 (40.3)	
MPO	91 (19.7)	2 (9.1)	89 (20.1)	
Negative	30 (6.5)	5 (22.7)	25 (5.7)	
Unavailable	151 (32.8)	1 (4.5)	150 (33.9)	
Comorbidities				
Diabetes	68 (14.8)	1 (5.3)	67 (15.2)	0.34
Hypertension	132 (28.6)	4 (21.1)	128 (29.0)	0.53
Chronic kidney disease	38 (8.2)	2 (10.5)	36 (8.1)	0.55
Chronic obstructive lung disease	7 (1.5)	0	7 (1.6)	0.70
Coronary artery disease	36 (7.8)	2 (1.05)	34 (7.7)	0.25
Sites of involvement (ever)				
Constitutional (*n* = 455)	367 (81.2)	12 (72.2)	354 (81.6)	0.23
Mucocutaneous (*n* = 453)	128 (28.4)	5 (29.4)	123 (28.4)	0.85
Musculoskeletal (*n* = 458)	225 (49.5)	8 (44.4)	217 (49.7)	0.55
Ocular (*n* = 456)	86 (19.0)	1 (5.9)	85 (19.5)	0.21
Ear–nose–throat (*n* = 457)	270 (59.5)	10 (58.8)	260 (59.5)	0.53
Respiratory (*n* = 461)	360 (78.6)	15 (83.3)	345 (78.4)	0.38
Vascular (*n* = 446)	23 (5.2)	0	23 (5.4)	0.29
Gastrointestinal (*n* = 455)	31 (6.9)	1 (5.6)	30 (6.9)	0.82
Genitourinary (*n* = 456)	252 (55.6)	13 (68.4)	239 (55.1)	0.21
Central nervous system (*n* = 456)	15 (3.3)	0	15 (3.4)	0.51
Peripheral nervous system (*n* = 445)	67 (15.2)	2 (11.1)	65 (15.3)	0.44
BVAS at baseline, median (IQR)	11 (11)	11 (15)	11 (11)	0.84°
Revised five-factor score ≥ 1 (*n* = 314)	206 (65.6)	7 (58.3)	199 (65.9)	0.77
Dialysis at baseline	33 (7.2)	3 (15.8)	30 (6.8)	0.15
Plasma exchange at baseline	54 (11.7)	1 (5.3)	53 (12.0)	0.82
Mortality	58 (12.6)	7 (36.8)	51 (11.5)	0.04

*According to the last follow-up visit

**p excluding the ‘‘unavailable’’ strata

°*p* values represent the result of the Mann–Whitney *U* test; all other *p* values represent the result of the log-rank test

ANCA serology (ELISA) differed between groups (PR3 and negative ANCA) and was more prevalent in patients with cancer, whereas MPO ANCA was more prevalent in patients without cancer (Table 1). Comorbidities (diabetes, hypertension, chronic kidney disease, chronic obstructive lung disease, and coronary artery disease) were similarly prevalent in both groups. Percentages of pulmonary involvement (83.3% vs. 78.4%, log-rank *p* = 0.38) [there was no significant difference regarding pulmonary involvement type between AAV patients with and without cancer] and renal involvement (68.4% vs. 55.1%, log-rank *p* = 0.21) were similar in both groups. Other sites of involvement, disease activity, prognostic scores, and the need for dialysis and plasma exchange at baseline were also comparable between groups (Table 1). Fifty-eight patients died during follow-up due to any cause: 7 (36.8%) in AAV patients with cancer and 51 (11.5%) in those without cancer (log-rank *p* = 0.04). Demographic and clinical characteristics of patients are shown in Table 1.

Patients with cancer were less likely to be exposed to rituximab than those without cancer (21.1% vs. 58.8%, log-rank *p* < 0.001). Both groups used other drugs in similar percentages (Table 2). The cumulative cyclophosphamide dose [median (minimum–maximum)] dose was 5.4 (0.5–30.0) g in AAV patients with cancer and 3.0 (1.5–7.0) g in those without cancer (*p* = 0.19).

**Table 2:** Distribution and time-dependent univariable comparison of treatments ever used in AAV patients with and without cancer (up to the time of cancer diagnosis or last follow-up visit)

Treatments	All patients (*n* = 461)	AAV patients with cancer (*n* = 19)	AAV patients without cancer (*n* = 442)	*P* value°
Methotrexate	76 (16.5)	1 (5.3)	75 (17.0)	0.08
Mycophenolate mofetil	58 (12.6)	1 (5.3)	57 (12.9)	0.42
Azathioprine	261 (56.6)	14 (73.7)	247 (55.9)	0.99
Cyclophosphamide	270 (58.6)	10 (52.6)	260 (58.8)	0.14
Azathioprine + cyclophosphamide	167 (36.2)	7 (36.8)	160 (36.2)	0.11
Rituximab	264 (57.3)	4 (21.1)	260 (58.8)	< 0.001
Pulse steroid at baseline	198/453 (43.7)	5/18 (27.8)	193/435 (44.4)	0.46
Sequential cyclophosphamide and rituximab (or vice versa)	263 (58.8)	3 (15.8)	260 (58.8)	< 0.001

*AAV* ANCA-associated vasculitis

°*p* values represent the result of the Mann–Whitney *U* test; all other *p* values represent the result of the log-rank test

**Fig. 1:**
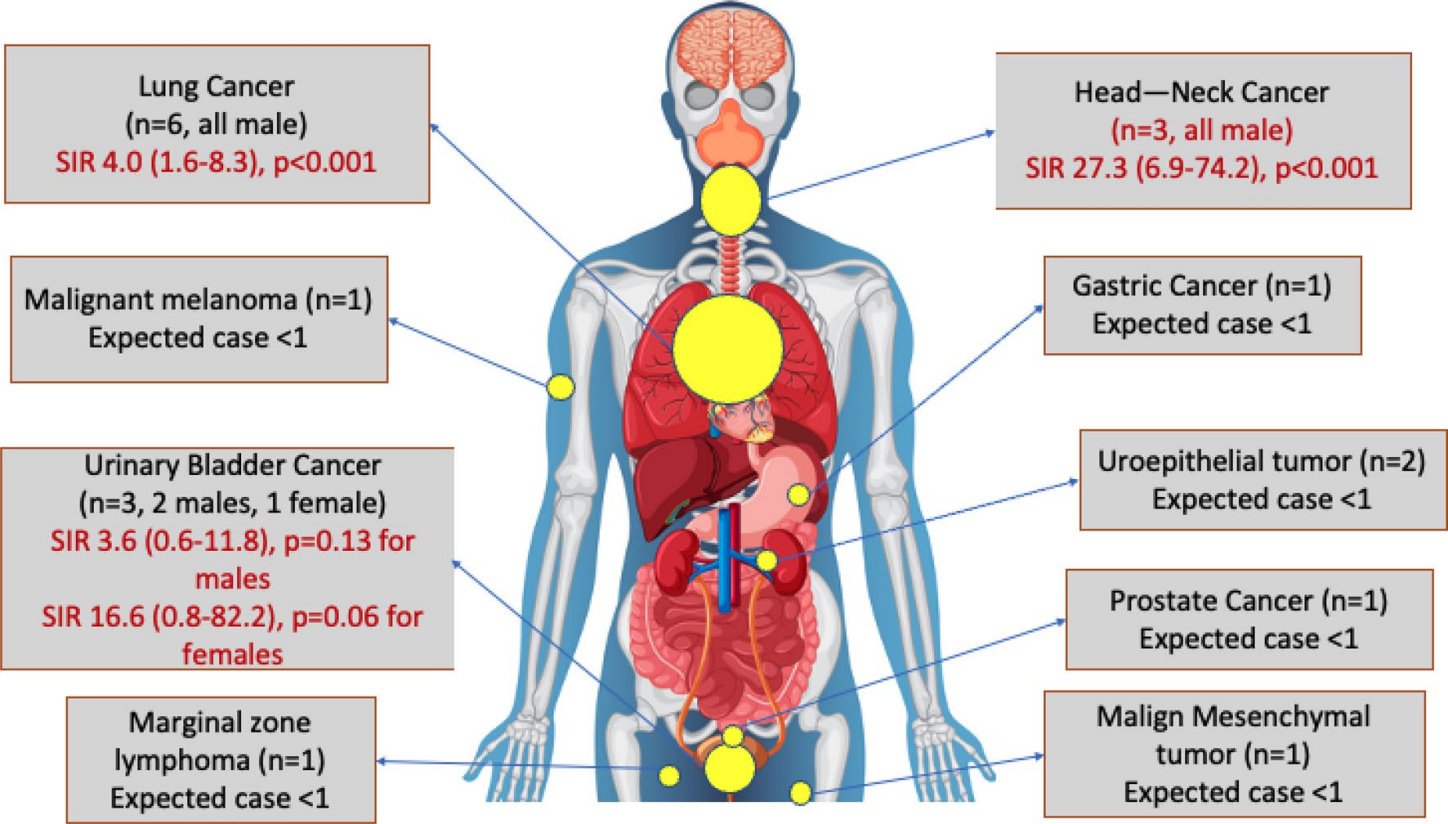
Distribution of cancer sites in AAV patients

Of 19 patients, 18 (94.7%) were male. Overall, cancer risk was higher in AAV patients compared to sex- and age-specific cancer risk of the general population (SIR 2.1 (1.3–3.2), *p* = 0.004)). The distribution of cancers in the schematic figure is given in Fig. 1. As the male sex was predominant, analyses were stratified according to sex.

i. In males
  Cancer risk was higher than the age-specific cancer risk of the male general population (SIR 3.1 (1.9–4.7), *p* < 0.001). Lung cancer was the most common cancer type (6 patients, SIR 4.0 (1.6–8.3), *p* < 0.001), followed by head–neck cancer (3 patients, SIR 27.3 (6.9–74.2), *p* < 0.001). Details of the other types of cancers are given in Table 3. According to age at AAV diagnosis, cancer SIRs were as follows: 2.3 (1.3–3.8, *p* = 0.008) for ≥ 50 years, 1.9 (0.95–3.3, *p* = 0.07) for ≥ 55 years, 2.2 (1.1–3.9, *p* = 0.03) for ≥ 60 years, 1.7 (0.7–3.5, *p* = 0.23) for ≥ 65 years, and 1.2 (0.3–3.1, *p* = 0.75) for ≥ 70 years.

**Table 3:** Standardized incidence rates according to cancer types of AAV groups

Subgroup	Total (*n*,%)	Observed cancer (*n*)	Expected cancer (*n*)	SIR	Confidence interval (95%)	*p* value
Overall	461 (100)	19	9.2	2.1	1.3–3.2	0.004
*Male*	236 (51.2)					
Overall		18	5.9	3.1	1.9–4.7	< 0.001
Lung		6	1.5	4	1.6–8.3	< 0.001
Head–neck SCC*		3	0.11	27.3	6.9–74.2	< 0.001
Urinary bladder		2	0.56	3.6	0.6–11.8	0.13
Prostate		1	1.03	0.97	0.05–4.78	0.99
Malign melanoma		1	0.04	25	1.2–123.3	0.04
Stomach		1	0.4	2.5	0.1–12.3	0.39
Non-Hodgkin lymphoma		1	0.15	6.7	0.3–32.9	0.14
Mesenchymal tumor		1	0.04	25	1.2–123.3	0.04
Uroepithelial tumor^++^		2	NA	NA	NA	NA
*Female*	225 (48.8)					
Overall		1	3.3	0.3	0.1–1.5	0.20
Urinary bladder		1	0.06	16.6	0.8–82.2	0.06

*Head–neck SCC cases were considered different from skin SCC by oncologists in these cases

^++^SIR was not calculated, as separate data were unavailable in the 2017 TNCR data for uroepithelial tumors

*SIR* standardized incidence rate
ii. In females
  Cancer risk was similar to the age-specific cancer risk of the female general population (SIR 0.3 (0.1–1.5), *p* = 0.20). There was one case with urinary bladder cancer (Table 3).
  Among patient subgroups, each of the following subgroups had significantly higher cancer incidence rates than their counterparts: male sex, age at ≥ 60 years at AAV diagnosis, and ever smoked (Table 4). Patients who received rituximab had a significantly lower cancer incidence rate than patients who did not receive rituximab (IRR 0.1 (0.03–0.4), *p* < 0.001) (Table 4).

**Table 4:** Incidence rates and incidence rate ratios according to subgroups of AAV patients

Subgroup	Total (*n*,%)	Observed cancer (*n*)	Follow-up (patient-years)	IR (95% CI) (per 1000 patient-years	IRR (95% CI)	p for IRR
AAV Subtype						
GPA	312 (67.7)	16	1442.7	11.1 (6.8–18.1)	2.1 (0.6–11.5)	0.22
Non-GPA	149 (32.1)	3	580.1	5.2 (1.6–16.0)	Reference	
ANCA status						
PR3	189 (61.0)	11	821.4	13.4 (7.5–24.1)	1.9 (0.4–17.6)	0.43
MPO	91 (29.4)	2	282.4	7.1 (1.8–28.2)	Reference	
Negative	30 (9.6)	5	137.4	36.4 (15.4–86.0)	5.1 (0.8–54.0)	0.07
Sex						< 0.001
Male	236 (51.2)	18	977.3	18.4 (11.7–29.1)	19.3 (3.0–802.3)	
Female	225 (48.8)	1	1045.5	1.0 (0.1–6.8)	Reference	
Age at diagnosis						< 0.001
< 60	337 (73.1)	8	1665.7	4.8 (2.4–9.6)	Reference	
≥ 60	124 (26.9)	11	357.1	30.8 (17.2–55.1)	6.4 (2.3–18.4)	
Smoking history						0.002
Never	239 (59.3)	5	1102.1	4.5 (1.9–10.8)	Reference	
Ever	164 (40.7)	14	688.9	20.3 (12.1–34.1)	4.5 (1.5–15.9)	
Pulmonary involvement						0.48
Yes	360 (78.6)	15	1511.7	9.9 (6.0–16.4)	1.6 (0.5–8.6)	
No	98 (21.4)	3	483.8	6.2 (2.0–19.2)	Reference	
Genitourinary involvement						0.21
Yes	252 (55.7)	13	1068.4	12.2 (7.0–20.8)	1.9 (0.7–5.9)	
No	201 (44.3)	6	919.0	6.5 (2.9–14.5)	Reference	
Immunosuppression, ever						
CYC, yes	270 (58.6)	10	1349.3	7.4 (4.0–13.7)	0.6 (0.2–1.5)	0.21
CYC, no	191 (41.4)	9	673.5	13.3 (7.0–25.6)	Reference	
AZA, yes	261 (56.6)	14	1454.7	9.6 (5.7–16.2)	1.1 (0.4–3.9)	0.89
AZA, no	200 (43.4)	5	568.1	8.8 (3.7–21.1)	Reference	
RTX, yes	264 (57.3)	4	1324.1	3.0 (1.1–8.0)	0.1 (0.03–0.4)	< 0.001
RTX, no	197 (42.7)	15	698.7	21.5 (13.0–35.4)	Reference	
MMF, yes	58 (12.6)	1	239.2	4.2 (0.6–29.6)	0.4 (0.01–2.6)	0.42
MMF, no	403 (87.4)	18	1783.6	10.0 (6.4–16.0)	Reference	
MTX, yes	76 (16.5)	1	408.6	2.4 (0.3–17.3)	0.2 (0.01–1.4)	0.10
MTX, no	385 (83.5)	18	1614.3	11.1 (7.0–17.7)	Reference	

*AZA* azathioprin, *CYC* cyclophosphamide, *GPA* granulomatosis polyangiitis, *IR* incidence rate, *IRR* incidence rate ratio, *MTX* methotrexate, *MPO* myeloperoxidase, *MMF* mycophenolate mofetil, *PR3* proteinase-3, *RTX*: rituximab
  Results of the univariable comparisons and Cox regression models are given in Supplementary Table 1. Due to the high rate of missing data, the ANCA ELISA variable was not included in the regression model. In the final model (results will be shown as hazard ratio (HR) and 95% confidence interval (95% CI)): *the male sex* 22.3 (2.9–170.2, *p* = 0.003), *age* ≥ *60 at AAV diagnosis* 7.1 (2.5–20.1, *p* < 0.001), and *receiving rituximab* 0.06 (0.01–0.26, *p* < 0.001) were independently associated with cancer risk.
  Individual characteristics of AAV patients with cancer are given in Supplementary Table 2.

## Discussion

In this multicenter study investigating 461 patients diagnosed with ANCA-associated vasculitis (AAV), the cancer risk in AAV patients was found to be 2.1 times higher compared to the age- and sex-specific cancer risk of the Turkish population. Lung cancer was the most frequently detected cancer type, while head and neck cancer incidence was also higher. Male sex and ≥ 60 years of age at AAV diagnosis were associated with an increased risk of cancer. No increased cancer risk was associated with using cyclophosphamide. However, using rituximab was associated with a decreased risk of cancer.

We found a greater cancer risk in males, despite the overall similar cancer risk reported for both sexes in the literature [3, 7, 8], which may be due to the genetic, hormonal, or risky behavioral differences between sexes or simply by detection bias. We also found higher cancer risk in AAV patients diagnosed at ≥ 60 years of age compared to younger patients, in addition to increased cancer risk in all age groups compared to the general population, especially in males. The late onset of AAV in cases with malignancy has been frequently reported in the literature [3, 7]. It is also plausible to speculate that patients diagnosed at an advanced age might be more susceptible to carcinogenesis that could develop due to the treatments they undergo, and their cumulative cancer risk might increase independently of AAV. Smoking is one of the most extensively studied and proven risk factors for cancer. However, data on smoking is lacking in many studies related to AAV and cancer. Although active or past smoking was more commonly found in cancer-diagnosed patients in our current study, it lost its significance in the multivariate model, and we did not see any interactions between smoking and age. Nevertheless, due to the abundance of missing data and the lack of information on the quantity and frequency of smoking, making interpretations on this topic within the scope of this study is challenging.

The relationships between AAV (anti-neutrophil cytoplasmic antibody-associated vasculitis) and other inflammatory rheumatic diseases with cancer have been extensively studied in specific disease contexts (rheumatoid arthritis OR 1.63 (1.23–2.15), dermatomyositis SIR 4.79 (3.71–5.87), and systemic sclerosis SIR 1.41 (1.18–1.68)) [9–12]. This relationship is mainly explained through the effects of inflammation on a cellular level and the drugs used in the treatment [13]. Inflammation can play a dual role in carcinogenesis, acting like a double-edged sword. It has been shown that the inflammasome-dependent cell death pathway called pyroptosis plays a role in eliminating lung, gastric, breast, hepatocellular, and colorectal cancer cells [13, 14]. On the other hand, chronic inflammation, such as interleukin 1 and TNF, has been directly associated with carcinogenesis [13, 15].

Aside from inflammation [14, 15], another factor associated with cancer is the drugs used in AAV treatment, especially cyclophosphamide [3, 16]. In the current study, cyclophosphamide was not associated with increased cancer risk. In addition, as confirmatory data to the current understanding of no association in our study, no patient used oral cyclophosphamide; all patients, received Mesna (sodium methanethiolate), for the prophylaxis of hemorrhagic cystitis and, more importantly, the cumulative cyclophosphamide dose was lower than in the earlier studies. Methotrexate and azathioprine may also increase the risk of skin cancer [17]. However, we excluded patients with skin cancer in our study. It is a relevant question to investigate whether immunosuppressives have similar effectiveness in patients with and without cancer, as this may be a surrogate for the role of chronic inflammation in carcinogenesis.

Regarding rituximab, recent data showed that the anti-tumor effect of rituximab is thought to originate from relatively increased anti-tumor cytotoxic T cells [18]. In addition, recent solid data from the UK suggested a lower risk of cancer in rituximab-treated compared to cyclophosphamide-treated patients. The cancer risk was comparable to the general population in rituximab-treated patients [19, 20]. Our results support the current literature about rituximab on cancer. However, we cannot entirely exclude the risk of selection bias regarding the use of rituximab, as clinicians might have prescribed rituximab to patients with a high risk of developing cancer with ‘sense de clinique’. For example, rituximab is the biological agent of choice for clinicians in rheumatoid arthritis patients with a history of cancer. This is because these agents are used as a treatment for hematologic malignancies themselves, and rituximab is one of the biological agents with the least negative effect on cancer [21]. So, inferring a causal relationship between rituximab and cancer is very complex. From a different perspective, despite the use of cyclophosphamide at a lower dose by current recommendations and the high rate of rituximab use in our cohort, the overall cancer risk was higher than in the general population.

Bladder cancer, related to the disease and cyclophosphamide usage, has been highlighted in numerous studies in the literature for increased incidence with pooled SIR of 3.8 (2.7–5.4) [3]. While the statistical significance of increased bladder cancer frequency based on sex stratification may not be apparent in our study, the presence of separately considered uroepithelial tumors can imply an elevated risk in genitourinary system cancers. However, the effect size of this risk is less substantial than in the literature, possibly due to the above-mentioned reasons. In the literature, although most studies regarding lung cancer do not report a statistically significant increase in risk, the meta-analysis, as mentioned above, presents an SIR value of 1.67 (1.07–2.60) for lung cancer [3]. Similarly, a large-scale population-based study conducted by Choi and colleagues utilizing propensity score matching has reported a significant increase in lung cancer risk [20].

Regarding low hematologic malignancy incidence in our cohort, the relatively low cumulative cyclophosphamide dose and the administration of rituximab in more than half of the patients may be the underlying reason. As we excluded patients with non-melanoma skin cancers from the outset of our study, no inferences can be drawn from the current data regarding this matter. Increased genitourinary, lung, and head–neck cancer risk in our study may be attributable to the fact that these tissues are the primary target for AAV, especially PR3-positive AAVs. Nevertheless, especially in patients with lung and head–neck involvement, the routine performance of lung and head–neck imaging may result in the more frequent and earlier detection of cancerous lesions. Consequently, an increased incidence might be observed due to the lead-time bias.

The multicenter nature, encompassing data from across the country, allowing for an exploration of the current short- to medium-term safety profile of immunosuppressive treatment in AAV, can be counted as the strength of our study. However, certain limitations must be acknowledged. The retrospective recording of some of the data from medical records introduces significant gaps, particularly in variables such as smoking, alcohol consumption, and environmental pollution, which could directly influence carcinogenesis. Lack of this kind of data may lead to overlooking possible associations and inferring incomplete associations. Moreover, notable missingness exists in the data regarding cumulative doses of cyclophosphamide, especially rituximab. Besides, we could not assess the cumulative dose of steroids and their effect on incident cancers. Although the distribution of major comorbidities was similar between the two groups, we cannot precisely estimate the impact of comorbidities on cancer occurrence due to the lack of severity data about comorbidities. Our study’s inability to address this lead-time bias risk is a noteworthy limitation. Lead time is the time between the early diagnosis of cancer and symptom occurrence, and lead-time bias takes place if early diagnosis of cancer may not cause a change in survival. Since these patients are followed and investigated more closely, tumoral lesions that may have a variable effect on survival may be detected early. In this case, diagnosing cancer that may not have a possible impact on survival may have caused us to find the incidence higher in AAV patients. It is known that cancer risk in rheumatic diseases is associated with increasing disease duration [22], and the cumulative follow-up period of the present study may be too short to reveal this relationship due to the small number of patients and the short follow-up period per person [23]. As is clearly stated in the study by Cuzick Field [20], longer follow-up studies are crucial to elucidating medical conditions. Since chronic inflammation is the most likely pathway to explain the association between AAV and cancer, analyzing patients exposed to this chronic inflammation for a longer period will help better understand the effect size of the factors discussed in this article. In addition, since the number of cancers detected will be higher in patients with longer follow-ups, the distribution between groups will be more homogeneous, and more stable regression models can be established. Also, SIR results should be interpreted cautiously due to the low number of cancer cases. Lastly, quantitative data regarding the smoking history was not available; therefore, we were only able to categorize smoking status as ever vs. never. It is well established that there is a clear dose–response relationship between active and long-term smoking and cancer, particularly lung cancer [24]. Due to the lack of quantitative data (i.e., pack-years), we cannot infer a relationship between cancer and the cumulative load of smoking, which may be a cause of incomplete modeling of the current study population. Considering that most cancers in the males were smoking-related cancers that were seen in the older ages, possible confounding due to smoking intensity could not be excluded.

In conclusion, we found 2.1 times higher cancer risk in AAV patients compared to the age- and sex-specific cancer risk of the male Turkish population. Lung and head-and-neck cancers were the most frequently detected cancer types. While no relationship could be established between increased cancer risk and cyclophosphamide usage, an association was identified between reduced cancer risk and the utilization of rituximab. Greater attention should be given to cancer risks, and screening programs should be effectively employed in male AAV patients and AAV patients diagnosed at advanced age. A more extended follow-up period for these patients has been planned. Furthermore, a global effort can be started under EUVAS and VCRC to clarify this concept better.

## Data Availability

Data is available upon request.
