# Correction: Unveiling cancer risk in ANCA-associated vasculitis: result from the Turkish Vasculitis Study Group (TRVaS)

**DOI:** 10.1007/s11739-024-03637-0

**Published:** 2024-05-30

**Authors:** Emre Bilgin, Tuba Demirci Yıldırım, Bahar Özdemir Ulusoy, Tahir Saygın Öğüt, Murat Karabacak, Öznur Sadioğlu Çağdaş, Reşit Yıldırım, Deniz Can Güven, Cansu Akleylek, Elif Ediboğlu, Muhammet Emin Kutu, Duygu Özgür, Rıza Can Kardaş, Ertuğrul Çağrı Bölek, Güllü Sandal Uzun, Zehra Özsoy, Emine Sarıyıldız, Gizem Ayan, Berkan Armağan, Abdulsamet Erden, Levent Kılıç, Funda Erbasan, Fatma Alibaz-Öner, Ebru Aşıcıoğlu, Ayten Yazıcı, Nazife Şule Bilge, Hamit Küçük, Selda Çelik, Cemal Bes, Servet Akar, Neslihan Yılmaz, Timucin Kaşifoglu, Ayse Cefle, Haner Direskeneli, Veli Yazısız, Ömer Dizdar, Ahmet Omma, Fatoş Önen, Ömer Karadağ

**Affiliations:** 1 Faculty of Medicine, Division of Rheumatology, Hacettepe University, Ankara, Turkey; 2 Hacettepe Vasculitis Research Center, Ankara, Turkey; 3 Faculty of Medicine, Division of Rheumatology, Dokuz Eylul University, İzmir, Turkey; 4 Division of Rheumatology, Ankara City Hospital, Ankara, Turkey; 5 Faculty of Medicine, Division of Rheumatology, Akdeniz University, Antalya, Turkey; 6 Faculty of Medicine, Division of Rheumatology, Marmara University, Istanbul, Turkey; 7 Faculty of Medicine, Division of Rheumatology, Kocaeli University, Kocaeli, Turkey; 8 Faculty of Medicine, Division of Rheumatology, Eskişehir Osmangazi University, Eskişehir, Turkey; 9 Division of Medical Oncology, Elazığ Fethi Sekin City Hospital, Elazığ, Turkey; 10 Faculty of Medicine, Division of Rheumatology, Demiroğlu Bilim University, Istanbul, Turkey; 11 Faculty of Medicine, Division of Rheumatology, Katip Çelebi University, İzmir, Turkey; 12 Division of Rheumatology, Bakırköy Sadi Konuk City Hospital, Istanbul, Turkey; 13 Division of Rheumatology, Başakşehir Çam Sakura City Hospital, Istanbul, Turkey; 14 Faculty of Medicine, Division of Rheumatology, Gazi University, Ankara, Turkey; 15 Faculty of Medicine, Division of Medical Oncology, Hacettepe University, Ankara, Turkey

**Correction: Internal and Emergency Medicine** 10.1007/s11739-024-03577-9

In this article one author name Abdulsamet Erden was incorrectly written as Abdussamet Erden.

The original article has been corrected.

